# Supplementary material for: Prediction Models and Decision Aids for Women with Ductal Carcinoma In Situ: A Systematic Literature Review
Source: Cancers (Basel). 2022 Jul 2;14(13):3259. doi: 10.3390/cancers14133259 (PMC9265509; doi:10.3390/cancers14133259)
Supplement: Supplementary file 1 [file cancers-14-03259-s001.zip › cancers-1794476-supplementary.pdf]

Table S1 search strategy

Search strategy

Medline(ovid)

N	SEARCHES
#3	1 and 2
#2	exp Decision Support Systems, Clinical/ or exp Decision Support Techniques/ or exp Decision Making, Computer-Assisted/ or Models, Theoretical/ or Risk Assessment/ or *Decision Making/ or (decision adj3 (support* or aid or aids or tool or tools or computer-aided)).ti,ab,kf. or ("decision making" or "theoretical model*" or "risk assess*" or "risk predict*").ti,ab,kf. or ((predict* or prognostic or diagnostic or decision* or risk*) adj3 (model* or outcome* or score* or factor* or rule* or variable* or scoring or analys* or tree*)).ti,ab,kf.
#1	exp Carcinoma, Intraductal, Noninfiltrating/ or (DCIS or "intraductal carcinoma*" or "ductal carcinoma in situ" or "atypical ductal hyperplasia*" or "in situ ductal carcinoma*").ti,ab,kf.

Embase(ovid)

N	SEARCHES
#3	1 and 2
#2	exp Decision Support Systems, Clinical/ or exp Decision Support Techniques/ or exp Decision Making, Computer-Assisted/ or Models, Theoretical/ or Risk Assessment/ or *Decision Making/ or (decision adj3 (support* or aid or aids or tool or tools or computer-aided)).ti,ab,kf. or ("decision making" or "theoretical model*" or "risk assess*" or "risk predict*").ti,ab,kf. or ((predict* or prognostic or diagnostic or decision* or risk*) adj3 (model* or outcome* or score* or factor* or rule* or variable* or scoring or analys* or tree*)).ti,ab,kf.

#1	exp Carcinoma, Intraductal, Noninfiltrating/ or (DCIS or "intraductal carcinoma*" or "ductal carcinoma in situ" or "atypical ductal hyperplasia*" or "in situ ductal carcinoma*").ti,ab,kf.

SCOPUS

N	SEARCHES
#1	(TITLE-ABS-KEY(dcis OR "intraductalcarcinoma*" OR "ductalcarcinomainsitu" OR "atypicalductalhyperplasia*" OR "insituductalcarcinoma*")) AND (TITLE-ABS((decision W/3 (support* OR aid OR aids OR tool OR tools OR computer-aided)) OR ("decisionmaking" OR "theoreticalmodel*" OR "riskassess*" OR "riskpredict*") OR ((predict* OR prognostic OR diagnostic OR decision* OR risk*) W/3 (model* OR outcome* OR score* OR factor* OR rule* OR variable* OR scoring OR analys* OR tree*))) OR AUTHKEY((decision W/3 (support* OR aid OR aids OR tool OR tools OR computer-aided)) OR ("decisionmaking" OR "theoreticalmodel*" OR "riskassess*" OR "riskpredict*") OR ((predict* OR prognostic OR diagnostic OR decision* OR risk*) W/3 (model* OR outcome* OR score* OR factor* OR rule* OR variable* OR scoring OR analys* OR tree*))))

TRIP

N	SEARCHES
#1	("ductal carcinoma in situ" OR DCIS OR "intraductal carcinoma" OR "atypical ductal hyperplasia" OR "in situ ductal carcinoma") AND ("decision support"~3 OR "decision aid"~3 OR "decision aids"~3 OR "decision tool"~3 OR "decision tools"~3 OR "computer aided decision"~3 OR "decision making" OR "theoretical model" OR "risk assess" OR "risk predict" OR "predict model"~3 OR "predict outcome"~3 OR "predict score"~3 OR "predict factor"~3 OR "predict rule"~3 OR "predict variable"~3 OR "predict scoring"~3 OR "predict analys"~3 OR "predict tree"~3 OR "prognostic model"~3 OR "prognostic outcome"~3 OR "prognostic score"~3 OR "prognostic factor"~3 OR "prognostic rule"~3 OR "prognostic variable"~3 OR "prognostic scoring"~3 OR "prognostic analys"~3 OR "prognostic tree"~3 OR "diagnostic model"~3 OR "diagnostic outcome"~3 OR "diagnostic score"~3 OR "diagnostic factor"~3 OR "diagnostic rule"~3 OR "diagnostic variable"~3 OR "diagnostic scoring"~3 OR "diagnostic analys"~3 OR "diagnostic tree"~3 OR "decision model"~3 OR "decision outcome"~3 OR "decision score"~3 OR "decision factor"~3 OR "decision rule"~3 OR "decision variable"~3 OR "decision scoring"~3 OR

	"decision analys"~3 OR "decision tree"~3 OR "risk model"~3 OR "risk outcome"~3 OR "risk score"~3 OR "risk factor"~3 OR "risk rule"~3 OR "risk variable"~3 OR "risk scoring"~3 OR "risk analys"~3 OR "risk tree"~3)

Table S2 IPDAS score Decision aids

**IPDAS checklist**	Berger Hoger et al. Score %	OnlineDecision.org Score %	DCISoptions.org Score %	Communication aid Score %
**I. Content: Does the patient decision aid …**				
**Provide information about options in sufficient detail for decision making?**	100	75	100	50
**Present probabilities of outcomes in an unbiased and understandable way?**	78	78	33	67
**Include methods for clarifying and expressing patients' values?**	67	0	33	0
**Include structured guidance in deliberation and communication?**	100	67	67	0
**II. Development Process: Does the patient decision aid …**				
**Present information in a balanced manner?**	100	50	50	0
**Have a systematic development process?**	86	86	14	86
**Use up to date scientific evidence that is cited in a reference section or technical document?**	50	33	0	50
**Disclose conflicts of interest?**	50	100	100	0
**Meet additional criteria if the patient decision aid is Internet based**	N.A.	40	33	N.A.
**Meet additional criteria if stories are used in the patient decision aid**	N.A.	N.A.	N.A.	N.A.
**III. Effectiveness: Does the patient decision aid ensure decision making is informed and values based?**				
**Decision processes leading to decision quality.**	100	75	75	50
